# Referral patterns and requested interventions in a cancer pain e-consult system

**DOI:** 10.1016/j.inpm.2026.100772

**Published:** 2026-05-17

**Authors:** Sarah Ahmed, Matthew Robert Smith, Moustafa Abdalla, Vicki Jackson, David Hao

**Affiliations:** a Department of Anesthesiology, Mass General Brigham, Harvard Medical School, Boston, MA, USA; b Department of Surgery, Mass General Brigham, Harvard Medical School, Boston, MA, USA; c Department of Medicine, Boston University Chobanian & Avedisian School of Medicine, USA

## Abstract

**Background:**

Chronic pain affects a substantial proportion of patients with cancer and remains undertreated despite advances in pharmacologic and interventional management. Electronic consultation (e-consult) systems have improved specialty access in other fields, yet their application to interventional cancer pain has not been described.

**Objectives:**

To characterize the referral patterns and requested interventions of a dedicated interventional cancer pain e-consult platform at a large academic medical center.

**Methods:**

We conducted a single-center retrospective study of interventional cancer pain e-consults placed through the electronic health record at Massachusetts General Hospital between January 1, 2020, and December 30, 2024. Consult orders were identified via structured EHR query and underwent manual chart review. Abstracted variables included demographics, referring specialty, intervention category, mortality status, and days from referral to death. Associations between intervention category and sex or mortality were evaluated using chi-square and Fisher's exact tests.

**Results:**

A total of 348 e-consults were included. Mean age at referral was 62 years (SD 15); sex distribution was near equal (49% male). At the time of review, 53% of patients were deceased, with a median interval from referral to death of 148 days (IQR 62–282). The most frequently requested interventions were celiac axis block (24%), spine-related procedures (16%), and opioid management (14%). Hospice and palliative medicine accounted for 53% of referrals, followed by gastrointestinal oncology (12%). Vital status at data extraction differed significantly by intervention category (p = 0.049), with celiac axis block associated with higher odds of deceased status (OR 1.76, p = 0.03) and intercostal nerve block associated with lower odds (OR 0.29, p = 0.04).

**Conclusions:**

A dedicated interventional cancer pain e-consult system attracted referrals spanning 19 procedural categories and multiple oncologic and palliative specialties, including a substantial proportion of patients near end of life. These findings suggest that e-consults represent a scalable, low-friction mechanism to broaden access to interventional pain expertise across the cancer care continuum.

## 1 Introduction

Chronic pain remains one of the most prevalent and burdensome symptoms experienced by patients with cancer. Approximately 39% of patients with cancer are impacted by cancer-related pain, with the prevalence increasing to 55% amongst those with active treatment and 66% in patients with advanced/metastatic disease [1]. Despite decades of therapeutic advancement, cancer-related pain continues to be undertreated. A 2023 systematic review examining the adequacy of analgesic therapy reported that approximately 44.5% of patients with cancer pain receive insufficient pain management (categorized as moderate to severe pain)[2]. Poorly controlled pain is associated with diminished physical functioning, increased psychological distress, impaired social participation, and reduced overall quality of life [3,4].

The global burden of cancer continues to grow, with 19.3 million new cases reported worldwide in 2020 and projections exceeding 28 million annually by 2040[5]. Advances in cancer detection and treatment have extended survival, resulting in a growing population of patients living with chronic and treatment-related pain. As survivorship increases, sustained and coordinated approaches to pain management that extend beyond acute or end-of-life care settings have become increasingly essential.

The management of cancer-related chronic pain has historically relied on multimodal strategies. The World Health Organization (WHO) analgesic ladder established the foundation for stepwise pharmacologic management, emphasizing appropriate opioid use for moderate to severe pain [6]. Contemporary guidelines from professional organizations, including the American Society of Clinical Oncology (ASCO), recommend an individualized approach incorporating opioid and non-opioid pharmacotherapy, adjuvant medications, regional and interventional procedures, alongside non-pharmacologic therapies such as physical therapy and behavioral interventions [7]. Interventional techniques including epidural steroid injections, radiofrequency ablations, and neurolytic blocks are particularly relevant for patients with inadequate pain control or intolerable side effects from pharmacologic management [8]. Despite their demonstrated value, access to specialty pain services remains inconsistent, often limited by geographic, institutional, and referral barriers(Elhakim et al., 2019)[9].

Electronic consultation (e-consult) systems have emerged as one strategy to address barriers in specialty access. E-consults are secure, asynchronous communication platforms that allow primary or referring clinicians to seek specialty input without requiring an in-person visit. Prior studies have demonstrated that e-consults can reduce wait times, improve access to specialty expertise, and enhance care coordination across disciplines[10,11]. Although the model has been successfully implemented in fields such as dermatology, cardiology, and psychiatry, its application in oncology, particularly in the management of chronic cancer-related pain, remains less well characterized[[12], [13], [14]].

Given the rising prevalence of chronic pain among patients with cancer, increasing survivorship, and the growing need for coordinated interdisciplinary care, structured e-consult systems represent a promising approach to improving pain management access and delivery. This study examines the utilization and impact of a dedicated interventional cancer pain e-consult platform, with particular attention to access to care and patterns of referral across specialties.

## 2 Methods

We conducted a single-center retrospective study of interventional cancer pain e-consult orders placed through the electronic health record (Epic Systems, Verona, WI) at Massachusetts General Hospital (MGH) between January 1, 2020, and December 30, 2024. The study was reviewed by the Mass General Brigham Institutional Review Board and deemed exempt (Protocol 2024P003411). E-consults were identified via structured query of the EHR, and each order underwent a manual chart review. This is a cross sectional analysis of e-consult requests as submitted by referring clinicians. Data on whether the requested interventions were ultimately performed were not abstracted and are outside the scope of this analysis.

The Cancer Pain Intervention E-Consult order within the MGH Epic electronic health record required the referring clinician to select a “Reason for E-Consult" either from a set of predefined intervention categories (celiac plexus block, intercostal nerve block, other nerve block, epidural/intrathecal drug delivery, or cryoablation) or through a free-text entry, and to complete a required free-text “Specific Patient Care Question"; an optional “Additional Comments" free-text field was also available. Embedded process instructions directed referring clinicians to use the e-consult only for specific, non-urgent clinical questions and to contact the MGH Pain Clinic directly at the listed phone number for in-person consultations. The interventional pain consultant then reviewed the patient's chart to determine candidacy for a cancer pain intervention and contacted the referring clinician within 48 h regarding next steps. Referring clinicians were therefore not obligated to commit to a specific intervention and could use the free-text fields to describe the clinical problem and defer intervention selection to the consultant.

Consult characteristics and clinical variables were abstracted and standardized, including demographics (sex, age at referral, race, and ethnicity), patient status at the time of review (deceased versus alive), referring specialty or service, and the requested intervention or clinical question. For deceased patients, the interval between e-consult referral and death was calculated in days.

Each e-consult was assigned to a single intervention category through manual review of the order summary, including the documented reason for consultation and specific patient care question. Cases were classified into predefined anatomic and procedural categories, including Celiac Axis Block, Spine, Epidural Steroid Injection, Intercostal Nerve Block, Intrathecal Drug Delivery, Cryoablation, and others, based on the primary clinical concern. Orders that could not be assigned were designated as Non-Specific Intervention. Ambiguous cases were resolved by consensus between reviewers. Where procedural volume and clinical specificity warranted, subcategories were designated independently; Celiac Axis Block was separated from other sympathetic interventions (grouped under Sympathetic Block), and Epidural Steroid Injection was separated from broader spine-related procedures.

Continuous variables are reported as mean (standard deviation) and median (interquartile range). Categorical variables are reported as counts and percentages. Pearson's chi-square test was used to evaluate associations between the intervention category and sex, as well as mortality. Exploratory subgroup analyses assessing the relationships between Celiac Axis Block and Intercostal Nerve Block with mortality status were performed using Fisher's exact test. Statistical significance was defined as a two-sided p-value of less than 0.05. All statistical analyses were performed using Python v3.14 [15]. No adjustment was made for multiple testing, and an a priori power calculation was not performed.

Artificial intelligence–assisted technology (Claude, Anthropic) was used during manuscript preparation for grammar review and sentence construction. It was not used for content generation, data analysis, or interpretation of results. All content was reviewed, verified, and approved by the authors, who bear full responsibility for the accuracy and integrity of the work.

## 3 Results

**Fig. 1:**
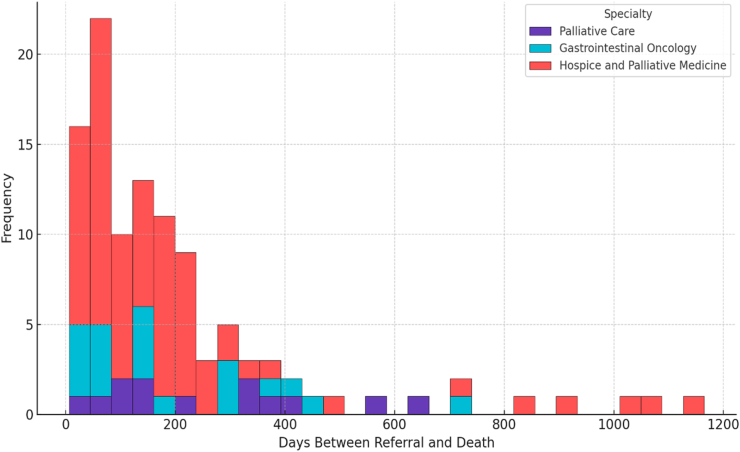
Distribution of days between referral and death among patients deceased prior to data extraction (Top 3 specialties).

A total of 348 interventional cancer pain e-consults were included in the analysis (Table 1). The mean age at referral was 62 years (SD 15), with a median of 64 years (interquartile range [IQR] 55–71). Sex distribution was near equal, with 172 males (49%) and 176 females (51%). The majority of patients identified as White (85%), with smaller proportions identifying as Black or African American (4%), Asian (4%), or American Indian/Alaska Native (0%; n = 1). Most patients were not Hispanic (94%) (Table 1). At the time of data extraction, 183 patients (53%) were deceased. Among deceased patients with available data, the median interval from e-consult referral to death was 148 days (IQR 62-282; range 4 to 1282), reflecting a wide spectrum of disease trajectory at the time of consultation (Fig. 1).

**Table 1:** Patient demographics and interventional cancer pain E-consult details.

	Total N = 348
Age at referral, years	61.7 (14.8)
Sex	
Male	172 (49%)
Race	
White	297 (85%)
Black or African American	15 (4%)
Asian	14 (4%)
American Indian or Alaska Native	1 (0%)
Ethnicity	
Not Hispanic	326 (94%)
Deceased at Time of Review	183 (53%)
Days from Referral to Death, Median (IQR)	148 (62-282)
Intervention Category	
Celiac Axis Block	84 (24%)
Spine	55 (16%)
Opioid management	48 (14%)
Epidural steroid injection	23 (7%)
Non-Specific Intervention	19 (5%)
Abdomen and Pelvis	17 (5%)
Intercostal Nerve Block	16 (5%)
Lower Extremity	15 (4%)
Other	17 (5%)
Cryoablation	6 (2%)
Intrathecal Drug Delivery	5 (1%)
Sympathetic Block	4 (1%)
Referring Specialty	
Hospice and Palliative Medicine	184 (53%)
Gastrointestinal Oncology	42 (12%)
Survivorship Program	18 (5%)
Genitourinary Oncology	16 (5%)
Other	88 (25%)

Data is presented as mean (standard deviation) or frequency (percentage) depending on variable type. Percentages have been rounded and may not total to 100%.

Other includes the following intervention categories: Head and Neck (n = 9), Chest (n = 9), Brachial Plexus (n = 10), Nerve Block — Specific (n = 10), Trigger Point Injections (n = 8), and Upper Extremity (n = 8).

Intervention utilization spanned a broad range of clinical categories. The most frequently requested intervention was Celiac Axis Block (n = 84), followed by Spine-related procedures (n = 55) and Opioid Management (n = 48). Other commonly represented categories included Epidural Steroid Injection (n = 23), Non-Specific Intervention (n = 19), Abdomen and Pelvis procedures (n = 17), Intercostal Nerve Block (n = 16), and Lower Extremity procedures (n = 15). Less frequently requested interventions included Cryoablation (n = 6), Intrathecal Drug Delivery (n = 5), and Sympathetic Block (n = 4).

Of the 348 included e-consults, 329 (95%) requested a specific intervention or management approach and were assignable to a defined category, whereas 19 (5%) were categorized as Non-Specific Intervention, reflecting referrals posing general cancer pain questions without an identifiable procedural request.

Sex distribution was balanced overall, though variation was observed across intervention categories. Brachial Plexus interventions were requested predominantly in females (80%), whereas Chest interventions (78%), Intrathecal Drug Delivery (80%), and Upper Extremity procedures (75%) were more frequently requested for male patients. There was no evidence of an association between sex and intervention category. Age at referral varied across intervention types. Patients referred for Epidural Steroid Injection had the highest median age (76 years; IQR 65–81), while those referred for Trigger Point Injections had the lowest (48 years; IQR 39–57).

Vital status at data extraction differed significantly by intervention category (χ^2^(17) = 27.64, p = 0.049). Higher proportions of patients who had died by the time of data extraction were observed among those consulted for Celiac Axis Block (63%), Cryoablation (67%), Nerve Block (60%), and Upper Extremity procedures (88%). Lower proportions were observed among those consulted for Chest interventions (22%), Intercostal Nerve Block (25%), Trigger Point Injections (25%), and Opioid Management (0.6%). In exploratory 2×2 comparisons without adjustment for multiple testing, Celiac Axis Block was associated with higher odds of death prior to data extraction (OR 1.76, p = 0.03), whereas Intercostal Nerve Block was associated with lower odds of death prior to data extraction (OR 0.29, p = 0.04).

Referral patterns revealed a concentration of consult volume among a limited number of specialties. Nearly half of all e-consults originated from Hospice and Palliative Medicine (53%), followed by Gastrointestinal Oncology (12%), Survivorship Program (5.2%), and Genitourinary Oncology (5%). Disease-specific referral patterns were also evident: Celiac Axis Block requests originated predominantly from Hospice and Palliative Medicine (57%) and Gastrointestinal Oncology (26%).

## 4 Discussion

E-consult systems have demonstrated benefit across multiple dimensions of care delivery, including enhanced access to specialty expertise, reduced wait times, cost savings, and educational value for referring clinicians. Among the most consistently reported advantages are improvements in access and timeliness, with e-consults enabling rapid specialist input and mitigating delays inherent to traditional face-to-face referral pathways. Prior studies also suggest meaningful reductions in downstream utilization, with one national sample reporting that approximately one in four e-consults avoided an in-person referral entirely or prevented referral to an inappropriate specialty[12,16,17]. Collectively, these findings support e-consults as an effective mechanism for delivering expert guidance while reducing friction in care pathways.

To our knowledge, this study represents the first examination of utilization patterns within a dedicated interventional cancer pain e-consult platform. Our findings characterize how such a platform is being used by referring clinicians caring for patients with cancer-related pain. The breadth of intervention categories represented in our cohort, 19 distinct procedural domains spanning commonly performed interventions such as celiac axis blocks to highly specialized therapies including intrathecal drug delivery, suggests that referring clinicians are using the platform to seek input across a wide spectrum of procedural complexity. This range indicates that the e-consult functions not merely as a triage tool for familiar procedures, but as a mechanism through which advanced interventional options can be introduced and considered within routine oncology and palliative workflows. Importantly, the present analysis did not examine whether e-consults obviated the need for in-person consultation, nor did it assess related downstream outcomes such as time to procedure, referral redirection, or procedure completion. These questions fall outside the scope of the current study and represent a focus of planned future work.

Several access-related themes also emerge from these data. First, there is a signal that many patients were near the end of life at the time of consultation, with a median interval from e-consult to death of approximately five months. For patients experiencing functional decline, traditional referral models often become impractical due to transportation challenges, appointment burden, and caregiver strain. In this context, asynchronous e-consultation offers a low-burden mechanism for specialty engagement, allowing clinicians to obtain expert input without requiring patients to navigate additional visits. The high proportion of referrals originating from hospice and palliative care services further underscores the relevance of this model for patients with advanced disease burden, in whom timely symptom-directed interventions may meaningfully impact quality of life.

Second, referrals originated from a wide range of specialties, including gastrointestinal oncology, survivorship programs, sarcoma, genitourinary oncology, radiation oncology, and palliative services, demonstrating the platform's ability to extend interventional pain expertise across diverse clinical contexts. This breadth suggests that the e-consult functions as a low-friction entry point for any clinician caring for patients with cancer-related pain to explore whether procedural options may be appropriate. Importantly, this structure also addresses a pervasive knowledge gap: many referring clinicians may be unaware of available interventional therapies or uncertain about indications. By enabling simple, asynchronous queries, the platform lowers the threshold for engagement and facilitates earlier consideration of interventional approaches. Notably, all Intrathecal Drug Delivery referrals originated exclusively from Hospice and Palliative Medicine, which may reflect either a concentration of awareness of this intervention or tendency to reserve this therapy for patients with advanced disease burden, or both.

Finally, our findings suggest that patients across the full spectrum of cancer trajectories, including those with active malignancy, advanced disease, and survivorship-related pain, may benefit from this model. While many patients were referred late in their disease course, the wide range in time from consult to death (spanning from days to several years) highlights that cancer-related pain is not confined to end-of-life care. Survivors frequently experience persistent pain related to prior surgery, radiation, chemotherapy, or structural sequelae of disease, and these patients were also represented within our cohort. This reinforces the importance of viewing interventional cancer pain services not solely as a terminal intervention, but as a longitudinal resource applicable throughout diagnosis, treatment, and survivorship.

## 5 Limitations

This study has several important limitations. Our analysis focuses on the utilization patterns of the e-consult platform and does not capture downstream clinical trajectories. Specifically, we do not have data on which patients ultimately underwent interventional procedures, time from e-consult to intervention, or post-procedure outcomes. As such, we cannot assess procedural conversion rates, treatment delays, or clinical effectiveness in this cohort. These endpoints represent important areas for future investigation.

Intervention categories were assigned through manual chart review, which included a mix of predefined selections and free-text entries. Several order categories lacked clinical specificity, and some referrals were inherently ambiguous, requiring reviewer interpretation. Although discrepancies were resolved by consensus, misclassification remains possible and reflects limitations of the original e-consult order structure.

This study also lacks a comparator group. We are therefore unable to quantify differences in access, timeliness, or utilization relative to traditional referral pathways. Although turnaround times for e-consults at our institution are typically measured in days, whereas in-person specialty referrals often occur over months, these observations are anecdotal and not formally evaluated.

Finally, this was a single-center retrospective study conducted at a large academic medical center with established interventional pain and palliative care services, which may limit generalizability to other practice settings. Referring clinicians at a large academic medical center with established interventional pain and palliative care services may have greater baseline familiarity with the range of available interventional therapies than clinicians in community or non-academic settings. The observed breadth of intervention categories and the distribution of referral sources may therefore overestimate the diversity of requests that would arise in settings with less established interventional pain infrastructure.

## 6 Conclusion

Taken together, these data support the interventional cancer pain e-consult as a scalable mechanism to improve access to specialized pain care, particularly for medically complex and geographically or functionally vulnerable patients. By providing a streamlined pathway for multidisciplinary engagement, this model has the potential to reduce delays, broaden awareness of interventional options, and integrate pain expertise more fully into oncology and supportive care workflows. Future work should examine patient-centered outcomes, referral conversion rates, and health system utilization to further define the clinical and operational impact of this approach.

## Declaration of interest

We have no conflicts of interest to report.
